# FACILITATORS OF BEHAVIORAL HEALTH CARE ACCESS AND UTILIZATION FOR TRANSGENDER OLDER ADULTS

**DOI:** 10.1093/geroni/igae098.1204

**Published:** 2024-12-31

**Authors:** Nik Lampe, Zena Rodill, Alexandra Nowakowski

**Affiliations:** University of South Florida, Tampa, Florida, United States; University of South Florida, Tampa, Florida, United States; Florida State University College of Medicine, Tallahassee, Florida, United States

## Abstract

Transgender older adults in the United States (US) experience significant disparities in behavioral healthcare access and utilization. However, the specific facilitators that contribute to positive or adequate behavioral healthcare experiences among this vulnerable aging population remain uncertain. Understanding the unique facilitators that shape positive behavioral healthcare experiences for transgender (trans hereafter) older patients can assist medical practitioners in integrating these lessons into their practices, thereby promoting cultural responsiveness in care. In this qualitative study, we aim to address the question: What factors facilitate positive behavioral healthcare access and treatment among transgender older adults in the US? Between September 2021 and January 2022, the first author conducted 47 semi-structured, individual interviews with transgender adults aged 65 or older residing in the US. Analyses focused on the behavioral healthcare access and utilization experiences of interview respondents. Data analyses were conducted collaboratively by the authors using NVivo (Release 1.6) software inductively using a grounded theory approach. Findings suggest opportunities for enhancing behavioral health support and resources for transgender older communities. Four main themes emerged from the data: (1) engaging with behavioral healthcare practitioners who offer compassionate, patient-centered care; (2) utilizing culturally tailored peer-support services; (3) ensuring equitable access to gender-affirming medical interventions; and (4) addressing financial and logistical barriers to behavioral healthcare access. These insights highlight opportunities for enhancing behavioral health support and resources for transgender older communities while emphasizing the importance of incorporating these factors into behavioral healthcare research and practice to promote cultural responsiveness in care for this vulnerable aging population.

